# Stone Milling of Dry Beans: Comparison with Hammer Milling, Physicochemical Characteristics of Flour, and Application in Wheat-Based Bakery Products

**DOI:** 10.3390/foods15214002

**Published:** 2026-11-07

**Authors:** Fatemeh Zare, Amrita Ray, Edil Vidal Torres

**Affiliations:** 1 Department of Nutrition and Food Sciences, University of Vermont, Burlington, VT 05405, USA; 2 Northern Crops Institute, Fargo, ND 58102, USA; amrita.ray@northerncrops.com (A.R.); edil.vidaltorres@ndsu.edu (E.V.T.)

**Keywords:** dry bean, stone milling, particle size, nutritional composition, damaged starch, pasting properties, sensory evaluation

## Abstract

Dry beans are nutrient-dense, sustainable ingredients, but the effects of milling mechanism on flour structure and food performance remain insufficiently understood. This study compared stone and hammer milling of black, navy, pinto, and red kidney beans to characterize differences in flour physicochemical properties and separately evaluate the application of stone-milled flours in wheat-based tortillas, tortilla chips, and crackers. Stone milling produced substantially greater damaged starch (0.820–1.169%) than hammer milling (0.277–0.400%), while particle-size responses varied by bean market class. Bean flours contained 22.96–24.90% protein and 17.55–18.10% dietary fiber. In wheat–bean blends, final viscosity increased from 574.5 cP in wheat flour to 786.5–1301.0 cP, with red kidney > navy > pinto > black bean. Sensory responses of products containing stone-milled bean flours were product-dependent; formulation significantly influenced selected appearance and texture attributes, while overall acceptance was generally comparable among formulations. These findings show that milling mechanism and bean market class jointly determine flour structure and functionality, supporting milling as a flour-design step for tailoring dry bean ingredients to specific wheat-based food applications.

## 1. Introduction

Dry beans (*Phaseolus vulgaris* L.) are nutritionally significant pulse crops that provide substantial amounts of plant protein, dietary fiber, complex carbohydrates, minerals, and other bioactive components. Their capacity for biological nitrogen fixation also contributes to their value within more sustainable agricultural and food systems [1,2,3]. Increasing interest in plant-based foods has expanded opportunities to use dry beans not only as whole-seed commodities but also as ingredients in value-added food products. Incorporation of bean-derived ingredients into cereal-based foods may therefore provide opportunities to improve nutritional density while expanding the utilization of pulse crops [1,2,4].

The nutritional value of dry beans is particularly relevant to cereal-based food applications because pulse proteins provide substantial amounts of lysine, an essential amino acid that is relatively limited in many cereal proteins. Conversely, pulse proteins are comparatively limited in sulfur-containing amino acids, making cereal–pulse combinations nutritionally complementary [5]. In addition to their nutritional contribution, pulse proteins and polysaccharides possess functional properties associated with water binding, emulsification, foaming, and gel formation making them useful functional ingredient for product application. However, the functionality of pulse flours depends not only on chemical composition but also on the physical structure generated during processing, including particle-size distribution, starch accessibility, starch damage, and interactions among starch, protein, and dietary fiber [5,6].

These characteristics have stimulated the incorporation of bean and other pulse flours into bakery and snack products. Pulse flour incorporation can increase protein and dietary fiber while modifying hydration, rheological behavior, texture, and other quality attributes of cereal-based products. Such effects arise partly from differences between pulse flours and wheat flour in protein functionality and carbohydrate composition [7,8]. Consequently, the functionality of pulse flour must be considered when developing cereal–pulse food systems because changes in physical properties of flour can affect both processing behavior and characteristics of the finished product.

Milling is an important upstream processing operation that influences the physical and functional characteristics of pulse flour. Unlike refined wheat milling, in which particle-size reduction is accompanied by systematic separation of anatomical components, whole-pulse milling simultaneously disrupts starch-, protein-, fiber-, and seed-coat-rich structures. The resulting flour is therefore a heterogeneous particulate system, and the extent of structural disruption can influence component accessibility, hydration, rheological behavior, and subsequent food performance [6]. These considerations become particularly important when whole-pulse or bean flour is incorporated into wheat-based products. Studies of pulse flours have demonstrated associations between particle size and functional properties such as water absorption, viscosity, and baking performance, although the optimal particle-size distribution may vary with pulse type and intended food application [9].

Starch damage is another important consequence of mechanical size reduction. Disruption of starch granules increases their accessibility to water and enzymes and can modify hydration and pasting behavior. Thus, damaged starch can serve as an indicator of the extent to which milling has altered the internal structure of the seed and can contribute to interpretation of subsequent flour functionality. Recent work has demonstrated relationships among particle size, starch damage, and pasting properties in pulse flours, although the magnitude and direction of these relationships depend on pulse type and flour structure [9,10]. Lee et al. [10], demonstrated that decreasing particle size increased damaged starch in several pulse flours and that coarse particles could also contribute substantially to pasting and gelling performance. These findings indicate that particle-size distribution cannot be interpreted solely as a measure of flour fineness; rather, it represents an important structural descriptor of pulse flour functionality.

The milling mechanism itself can further influence these structural characteristics. Size reduction may involve combinations of impact, compression, shear, abrasion, and cutting, with their relative contributions depending on mill design and operating conditions [6]. Stone and hammer mills provide contrasting mechanical systems that can generate different particle-size distributions and degrees of structural disruption. Evidence from black bean flour indicates that milling method affects particle size and other physical properties, while recent work with pinto bean has directly demonstrated differences among milling systems in starch damage and starch functionality [11,12]. Pasumarthi et al. [12] reported that hammer milling generated fine pinto bean flour with relatively low starch damage, whereas stone and burr milling produced higher starch damage. These findings support the interpretation that milling mechanism can generate functionally meaningful structural differences in pulse flours.

Despite increasing recognition of milling as a determinant of pulse flour functionality, the relationships among milling mechanism, bean market class, and resulting flour characteristics remain insufficiently characterized. Previous studies have investigated the effects of milling conditions on the distribution of starch-, protein-, and fiber-rich fractions in legumes [13], as well as the influence of particle size and milling history on pulse flour functionality [6,9]. However, systematic comparisons of stone and hammer milling across multiple dry bean market classes, particularly those integrating particle-size distribution and starch damage, remain limited. Furthermore, understanding how these milling-induced differences relate to flour functionality and potential food applications is important for selecting appropriate milling conditions and bean flours. Addressing these knowledge gaps could support a more systematic approach to optimizing dry bean flour production for specific food applications.

This relationship is particularly relevant when comparing different dry bean market classes because genotype and seed structure may influence their response to mechanical size reduction. Differences in seed hardness, anatomical structure, and the relative distribution of starch, protein, fiber, and seed-coat material can influence fracture behavior and the resulting particle-size and starch-damage profiles [9,13]. Therefore, a milling system (i.e., stone or hammer milling) that generates a particular structural response in one bean type may not produce an equivalent response in another. Evaluating multiple market classes under comparable milling conditions provides an opportunity to distinguish effects associated with milling mechanism from inherent differences among bean types. Stone milling is known for producing wholesome nutrition flour with a better uniform particle-size distribution and offers greater control over flour characteristics, but it may generate more heat and cause greater starch damage, depending on operating conditions. In contrast, hammer milling offers high throughput and efficient size reduction but may produce a broader particle-size distribution and less uniform flour. Therefore, study on milling methods to explore different bean flour functionality, processing efficiency, and intended food application is of utmost significance.

The four dry bean market classes selected for this study differ in their physical appearance and typical seed morphology. Navy beans are generally small and oval, black beans are typically small to medium-sized and oval, pinto beans are commonly medium-sized with mottled seed coats, and red kidney beans are generally larger and kidney-shaped. Although these classes belong to the same species, variation in seed dimensions, shape, and seed-coat characteristics may influence stress distribution and crack propagation during mechanical size reduction. Their internal structure, moisture content, and hardness may also contribute to differences in fracture behavior. Comparing these market classes therefore provides an opportunity to investigate whether the effects of milling mechanism on particle-size distribution and starch damage are consistent across beans with contrasting physical characteristics. Tortillas, tortilla chips, and crackers were selected as complementary wheat-based product models because they represent thin, relatively low-volume food matrices produced under distinct processing conditions. Unlike yeast-leavened bread, these products do not rely on gas retention and loaf-volume development as primary determinants of structure. Tortillas provide a model for evaluating dough handling and flexibility, whereas tortilla chips and crackers enable assessment of flour incorporation under frying and baking conditions, respectively. Evaluating these products therefore allows the technological relevance of bean flour characteristics to be examined across different product structures and thermal-processing conditions.

Accordingly, this study compared stone and hammer milling of black, navy, pinto, and red kidney beans to characterize the resulting particle-size distributions and starch damage. The functional properties of stone-milled bean flours were further evaluated in wheat-based composite systems through pasting analysis and application in tortillas, tortilla chips, and crackers. We hypothesized that (1) milling mechanism would significantly affect particle-size distribution and starch damage, and that these effects would differ among bean classes because of differences in seed morphology and physical structure; (2) particle-size distribution and starch damage would be associated with differences in flour pasting behavior; and (3) the functional characteristics of stone-milled bean flours would influence the quality of the resulting food products in a product-dependent manner. These hypotheses were evaluated to clarify the relationships among bean class, milling mechanism, flour characteristics, and technological performance.

## 2. Materials and Methods

### 2.1. Materials

Four commercially important dry bean (*Phaseolus vulgaris* L.) market classes—black, navy, pinto, and red kidney beans—were obtained from the dry edible bean breeding program at North Dakota State University. Beans were cleaned using a Carter Day dockage tester (Carter Day International, Minneapolis, MN, USA) to remove foreign materials and stored in airtight containers at ambient temperature until milling.

Commercial refined all-purpose wheat flour was obtained from a local supplier and used as the wheat-based component for composite flour preparation. Food-grade ingredients used for bakery-product preparation, including shortening, vegetable oil, salt, and baking powder, were purchased from commercial sources and used as received.

### 2.2. Bean Flour Preparation and Milling

**Figure 1:**
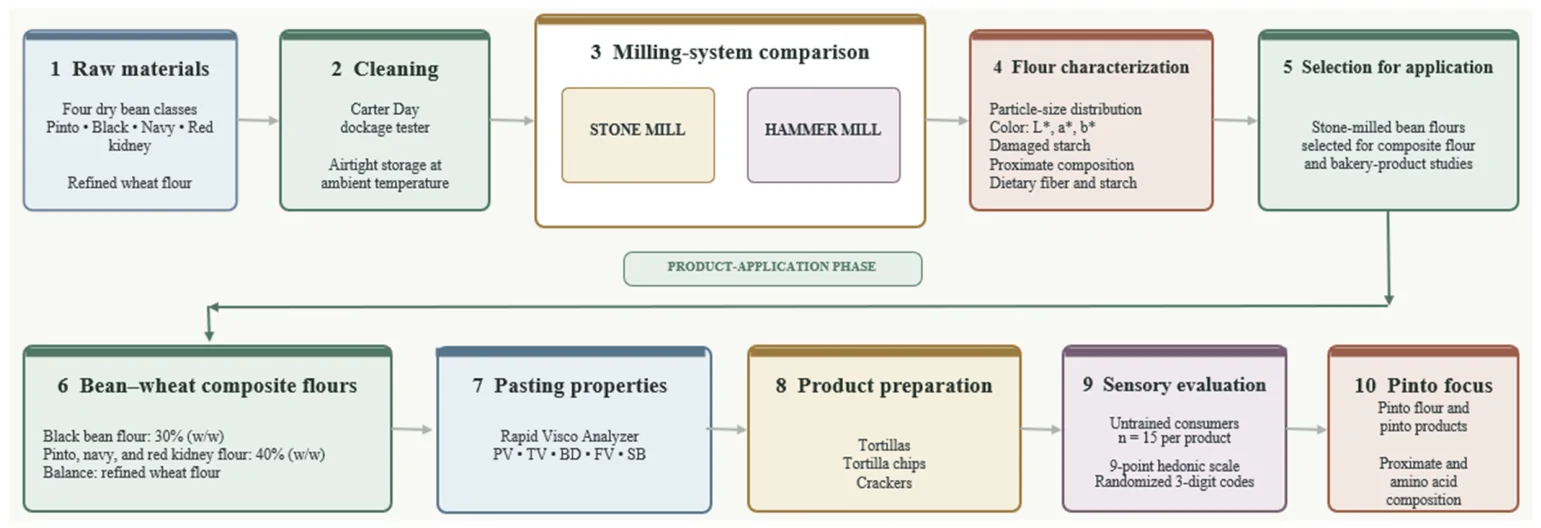
Experimental workflow for bean flour characterization and product development.

Figure 1 demonstrates the experimental workflow for bean flour characterization followed by product development. Cleaned dry beans were milled using two milling systems, stone milling and hammer milling, to evaluate the influence of milling technology on flour characteristics. Milling conditions were selected based on preliminary milling trials and previous research describing the effects of milling operations on particle-size distribution, structural disruption, and functional properties of pulse ingredients [13,14].

Stone milling was performed using a 26-inch New American Stone Mill (New American Stone Mills, Stowe, VT, USA). Preliminary trials were conducted to establish appropriate operating conditions for each bean type. Milling parameters, including stone-gap setting, rotational speed, and feed rate, were adjusted to obtain consistent flour production and desirable flour characteristics. Final conditions were selected based on milling performance, flour appearance, and preliminary observations of particle-size distribution. Stone gap was adjusted using the mill’s numerical readout, which represents a relative adjustment setting rather than a calibrated physical distance between the stones. Black and navy beans were milled using a narrow stone-gap setting (mill hand-dial marked at 300), a stone rotational speed of 208 rpm, and a feed rate of approximately 35 lb/h. Pinto beans were milled using a relatively wider stone-gap setting (mill hand-dial marked at 280), a rotational speed of 208 rpm, and a feed rate of approximately 30 lb/h. Red kidney beans were milled using a narrow stone-gap setting (mill hand-dial marked at 300), a rotational speed of 208 rpm, and a feed rate of approximately 30 lb/h.

Hammer milling was performed using a Fitz Mill (The Fitzpatrick Company, Elmhurst, IL, USA) using a two-pass procedure. The first pass was conducted using screen 1531 (0065), followed by a second pass using screen 1532 (0020). For each bean type and milling method, milling was conducted in two independent runs. Milling was conducted under ambient laboratory conditions (room temperature); however, flour temperature during and immediately after milling was not monitored. Following milling, flour from each run was collected separately, homogenized, transferred to airtight containers, and stored at room temperature until analysis.

### 2.3. Characterization of Bean and Wheat Flours

#### 2.3.1. Particle-Size Distribution Measurement

Particle-size distribution of bean flours was determined using a Ro-Tap^®^ Testing Sieve Shaker (Model B; Tyler Combustion Engineering, Inc., Mentor, OH, USA) according to AACC Method 55-60.01 [15].

Flour samples were fractionated through a series of standard sieves, and the mass retained on each sieve was recorded. Particle-size distribution was expressed as the percentage of material recovered within each mesh-size fraction relative to the total sample mass.

For particle-size analysis, two independent milling runs were evaluated for each milling method and bean type.

#### 2.3.2. Color Measurement

Color characteristics of bean flours were measured using a portable colorimeter (Minolta CR-410, Konica Minolta, Ramsey, NJ, USA). Measurements were recorded using the CIELAB color system, in which L* represents lightness, a* represents redness (+)/greenness (−), and b* represents yellowness (+)/blueness (−), following established color-measurement principles [16]. Three replicate measurements were obtained for each sample, and mean values were used for statistical analysis.

#### 2.3.3. Damaged Starch Determination

Damaged starch content of bean flours was determined using a Megazyme K-SDAM, assay kit (Megazyme Ltd., Bray, Co. Wicklow, Ireland), based on the AACC Method 76-31.01 [15].

Three replicate determinations were performed for each bean flour sample. Results were expressed as percentage damaged starch on a dry-matter basis.

#### 2.3.4. Proximate Composition, Dietary Fiber, and Starch Measurements

Proximate composition, and starch content of the bean flours and refined wheat flour were determined by an accredited external analytical laboratory using validated analytical procedures and applicable AOAC International methods [17]. Moisture, protein, fat, ash, total dietary fiber, and starch were determined using the laboratory’s validated procedures. Protein content was reported as crude protein using a nitrogen conversion factor of 6.25. Analytical results were reported as provided by the laboratory and were used for subsequent statistical analysis as described in Section 2.9.

### 2.4. Preparation of Bean–Wheat Composite Flour Blends

Stone-milled bean flours were used for composite flour preparation by replacing a targeted portion of refined wheat flour with bean flour at the substitution levels. Preliminary formulation trials were conducted to determine suitable bean flour incorporation levels for subsequent bakery-product development. Bean–wheat flour formulations were evaluated based on dough handling characteristics, processing performance, product appearance, and preliminary sensory observations. Based on these evaluations, black bean flour was incorporated at 30% (*w*/*w*), while pinto, navy, and red kidney bean flours were incorporated at 40% (*w*/*w*), with the remaining proportion consisting of refined wheat flour. The bean and wheat flours were weighed according to the selected substitution levels and thoroughly mixed to obtain homogeneous composite flour blends for subsequent analysis and product development.

### 2.5. Preparation of Bean-Based Bakery Products

Bean-based bakery products, such as tortillas, tortilla chips, and crackers, were prepared using the selected bean–wheat composite flour formulations. For each product, formulations were prepared using a 100-g composite flour basis.

#### 2.5.1. Tortilla Preparation

Tortillas were prepared using composite flour blends containing 30% black bean flour or 40% pinto, navy, and red kidney bean flour as wheat-flour replacements. For each formulation, 100 g of composite flour was mixed with baking powder (1.5 g), salt (1.0 g), shortening (30 g), and approximately 50 mL water to form a homogeneous dough. The dough was kneaded for 3–5 min and allowed to rest at room temperature for 20–30 min. A 50-g portion of dough was formed into tortilla sheets using a DoughPro DP2000 dough press (ProProcess Corporation, South Gate, CA, USA; 240 V, 3000 W, 50/60 Hz) equipped with Teflon-coated plates. The dough was pressed at approximately 90 °C for 10 s to obtain circular tortilla discs with a final thickness of approximately 1.5 mm. The formed tortillas were subsequently cooked on a preheated griddle for approximately 30–45 s per side until fully set and light-brown surface spots developed. Cooked tortillas were cooled to room temperature before subsequent analysis and sensory evaluation.

The formed tortillas were subsequently cooked on a preheated griddle for approximately 30–45 s per side until fully set and light-brown surface spots developed. Cooked tortillas were cooled to room temperature before analysis and sensory evaluation.

#### 2.5.2. Tortilla Chip Preparation

Tortilla chips were prepared using the same bean–wheat composite flour formulations.

For each formulation, 100 g of composite flour was mixed with salt (2 g), vegetable oil (15 g), and approximately 50–60 mL water to produce a firm dough. The dough was rested for 15–20 min, sheeted to approximately 1–2 mm thickness, and cut into standardized chip triangle pieces. The pieces were fried in vegetable oil at approximately 175–180 °C for 1–2 min until crisp and golden. Chips were removed from the oil, allowed to drain, cooled to room temperature, and stored in sealed containers until analysis and sensory evaluation.

#### 2.5.3. Cracker Preparation

Nonfermented crackers were prepared using the selected bean–wheat composite flour formulations. For each formulation, 100 g of composite flour was mixed with salt (2 g), vegetable oil (8 g), and approximately 40–50 mL water to obtain a firm dough. The dough was rested for approximately 10 min, sheeted to approximately 1–2 mm thickness, docked, and cut into standardized cracker pieces. Crackers were baked in a pizza oven at approximately 250–300 °C for 1–3 min until dry and lightly browned. Products were cooled to room temperature and stored before analysis and sensory evaluation.

### 2.6. Pasting Properties of Composite Flours

Pasting properties of wheat flour and bean–wheat composite flour blends were evaluated using a Rapid Visco Analyzer (RVA Super 4, Newport Scientific Pty Ltd., Warriewood, NSW, Australia) according to the manufacturer’s recommended procedure and standard RVA methodology [18]. A flour suspension was prepared by dispersing 3.0 g of sample (adjusted to a 14% moisture basis) in 25 mL of distilled water in an RVA canister. The suspension was mixed using the RVA paddle, and viscosity changes during the programmed heating and cooling cycle were recorded using Thermocline software (version 2.2, Newport Scientific Pty Ltd., Warriewood, NSW, Australia).

Peak viscosity (PV), trough viscosity (TV), breakdown viscosity (BD), final viscosity (FV), and setback viscosity (SB) were recorded.

Two replicate measurements were performed for each flour formulation, and mean values were used for statistical analysis.

### 2.7. Sensory Evaluation

The sensory evaluation involving human participants was reviewed and determined to be exempt from further review by the North Dakota State University Institutional Review Board (NDSU IRB) under Category 6 of the federal regulations for the protection of human subjects (45 C.F.R. Part 46). The study was approved under Protocol No. IRB0004629, titled Development of dry bean-based bakery products from stone milled flours, with an approval date of 17 January 2023. Participants provided informed consent prior to participation in the sensory evaluation.

Sensory evaluation was conducted using an untrained consumer panel to assess the acceptability of the developed bean-based crackers, tortillas, and tortilla chips. Fifteen panelists evaluated the products within each product category.

Samples were identified using random three-digit codes and presented in randomized order to minimize presentation-order effects. Panelists evaluated aroma, appearance, texture, flavor, and overall acceptability using a nine-point hedonic scale ranging from 1 = dislike extremely to 9 = like extremely, following the procedure described by Peryam and Pilgrim (1957), with slight modifications [19].

Sensory evaluations were conducted under controlled conditions. Water was provided for palate cleansing between samples. Data were collected electronically using Compusense Cloud sensory evaluation software (version 24.0.46 Compusense Inc., Guelph, ON, Canada).

### 2.8. Proximate and Amino Acid Composition of Pinto Bean Flour and Products

Pinto bean formulations were selected for additional nutritional characterization because pinto beans represent an important commercial dry bean market class in the United States. Accordingly, proximate composition and amino acid composition were determined for pinto bean flour and pinto bean-based bakery products. Proximate composition of pinto bean flour and pinto bean-based bakery products was measured according to the method described in Section 2.3.4, and amino acid composition was determined by an external analytical laboratory using validated amino acid analysis procedures based on the Official Methods of Analysis of AOAC International [19]. Amino acid concentrations were reported on a sample basis as mg/g sample. Amino acid composition was determined using a single analytical determination per sample (*n* = 1). The results were used for descriptive compositional characterization; therefore, standard deviations and statistical comparisons were not calculated for amino acid data. Essential amino acids (EAA) were calculated as the sum of threonine, valine, isoleucine, leucine, phenylalanine, lysine, histidine, and methionine. Branched-chain amino acids (BCAA) were calculated as the sum of valine, isoleucine, and leucine. Sulfur-containing amino acids (SAA) were calculated as the sum of methionine and cystine.

### 2.9. Statistical Analysis

Statistical analyses were performed using JASP statistical software (Version 0.98.1; JASP Team, 2026, Amsterdam, the Netherlands). Data are presented as mean ± standard deviation (SD), and statistical significance was set at *p* < 0.05.

Bean-flour color and damaged-starch content were analyzed using two-way analysis of variance (ANOVA), with bean type (black, navy, pinto, and red kidney), milling method (stone and hammer), and their interaction as fixed effects. Three replicate observations were analyzed for each bean type × milling method combination. Significant effects were followed by Tukey’s honestly significant difference (HSD) test for multiple comparisons.

Particle-size distribution was evaluated separately for each bean type using two independent milling runs per milling method. Stone- and hammer-milled flours were compared within individual mesh classes using Welch’s two-sample t test, with *p* values adjusted using the Benjamini–Hochberg false discovery rate (FDR) procedure. Differences in overall particle-size distributions between milling methods were additionally assessed using an exact permutation test for each bean type. Given the limited number of independent milling runs (*n* = 2 per method), particle-size analyses were considered exploratory.

Proximate composition, starch, and dietary fiber were analyzed using one-way ANOVA with flour type as the fixed factor. Pasting parameters (PV, TV, BD, FV, and SB) were similarly analyzed using one-way ANOVA with flour formulation as the fixed factor. Significant effects were followed by Tukey’s HSD test.

Sensory attributes (aroma, appearance, texture, flavor, and overall acceptability) were analyzed separately within each product category using one-way repeated-measures ANOVA, with product formulation as the within-subject factor and panelist as the repeated-measures subject. Significant formulation effects were followed by paired comparisons with Holm adjustment for multiple comparisons. Sensory analyses included 15 panelists per product category.

Exploratory Spearman rank correlation analysis was performed to examine associations among particle-size fractions, damaged starch, RVA pasting parameters, and sensory attribute scores. Correlations between particle-size distribution and damaged starch were evaluated using treatment means from the eight-bean class × milling method combinations. Associations involving RVA parameters and sensory attributes were examined separately using the four stone-milled bean formulations for which corresponding measurements were available. Sensory attributes were represented by mean panel scores for each formulation and product category. The wheat-only control was excluded because corresponding bean flour characteristics were unavailable. Given the limited number of independent formulations and differences in bean flour incorporation levels, correlations were interpreted descriptively rather than as confirmatory statistical evidence.

Amino acid composition was determined by an external analytical laboratory using a single analytical determination per sample. Because replicate measurements were unavailable, amino acid data were reported descriptively without SD or inferential statistical comparisons.

## 3. Results and Discussion

### 3.1. Flour Color Characteristics

Data shown in Table 1 showed that navy bean flour was substantially lighter than the other bean flours, with L* values of 67.99–68.73, compared with 52.42–52.68 for pinto, 28.81–30.45 for red kidney, and 24.93–25.11 for black bean flour. This ranking was consistent with the visual pigmentation of the respective seeds. The relatively small differences in L* between stone- and hammer-milled samples indicate that milling did not substantially alter overall flour lightness. Similar effects of milling method on black bean flour color and other physical properties have been reported previously [11]. The a* values provided greater differentiation among the market classes. Red kidney bean flour exhibited the highest positive a* values (18.64–19.46), consistent with its characteristic red pigmentation, whereas black and navy bean flours had values close to neutrality. Pinto bean flour showed an intermediate positive a* response (6.97–8.41). The significant interaction between bean type and milling method indicates that the effect of milling on redness depended on the bean material. For example, hammer milling increased a* in pinto bean flour but decreased it in red kidney bean flour. Such differences may reflect changes in the relative exposure and distribution of pigmented seed-coat and cotyledon material during particle fracture; however, because anatomical fractions were not separately analyzed, this interpretation remains inferential. The b* values also varied among bean types, with pinto bean flour exhibiting the highest positive values and black and red kidney bean flours showing substantially lower values. Hammer milling increased b* in pinto and navy bean flours but had comparatively little effect on red kidney bean flour.

Bean type (market class) significantly affected all color parameters (*p* < 0.001), whereas milling method had a smaller effect, with a significant effect observed only for b* (Table 1). Significant bean type by milling method interactions were detected for a* and b*, indicating that milling effects on these color attributes varied among bean types. Overall, flour color was primarily determined by the inherent pigmentation and anatomical characteristics of the beans, while milling produced more limited, bean-dependent changes. Thus, the differences in flour color observed among bean types largely reflected raw-material characteristics rather than processing effects, an important consideration for subsequent product evaluation.

### 3.2. Particle-Size Distribution

**Figure 2:**
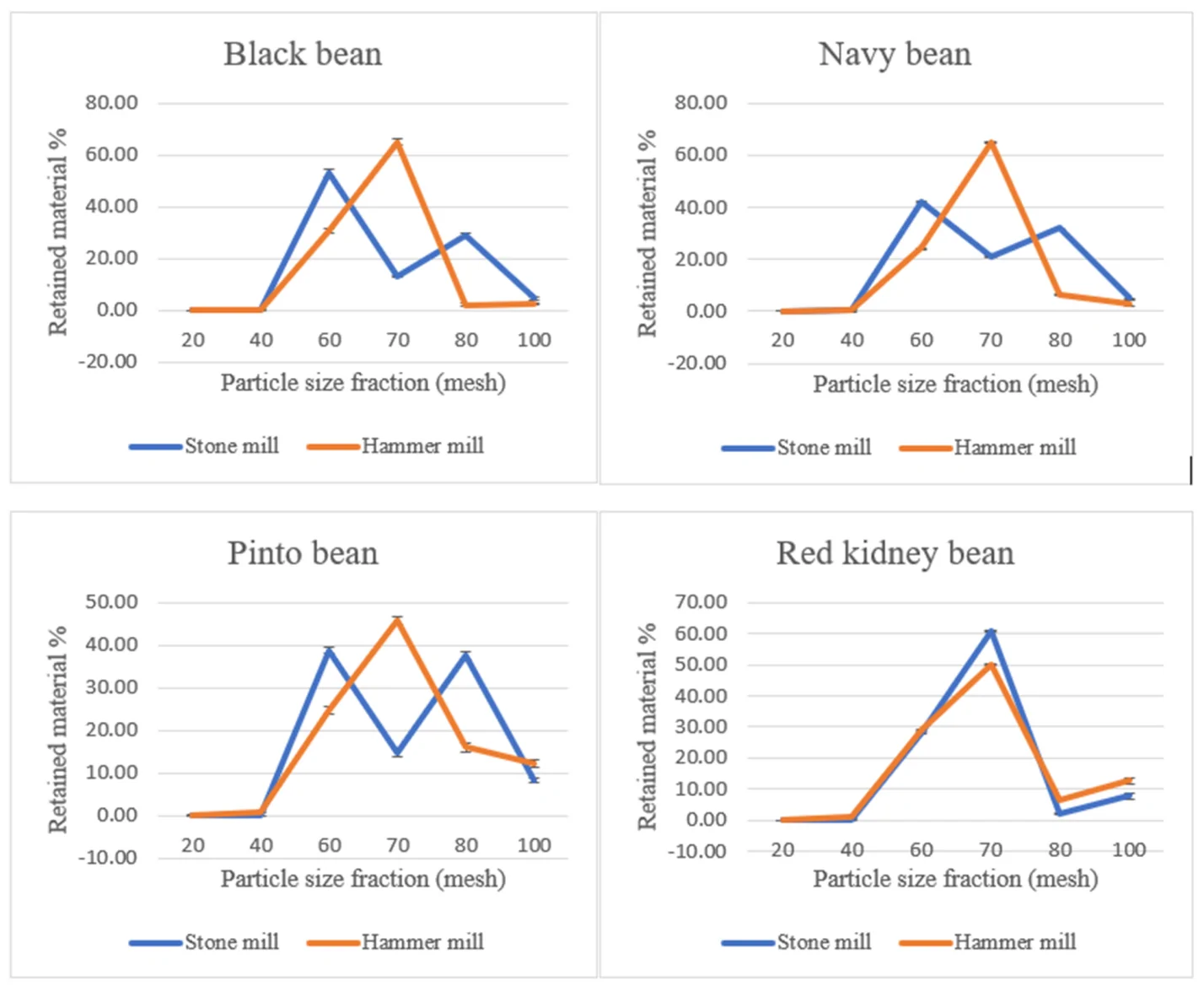
Particle-size distribution of stone- and hammer-milled beans flour from four *Phaseolus vulgaris* L. market classes (black, navy, pinto, and red kidney bean). Values represent the mean percentage of material recovered in each mesh fraction from two independent milling runs per milling method; error bars represent standard deviation (SD).

The two milling systems produced distinct particle-size profiles, but the magnitude and pattern of the response depended strongly on bean market class (Figure 2). The clearest separation between milling treatments occurred within the 60–80 mesh range for black, navy, and pinto beans, whereas red kidney bean exhibited a comparatively limited response. These findings indicate that milling method affected not only the overall fineness of the flour but also the distribution of particles among size classes. Such treatment-dependent differences are expected for pulse flours because particle breakage is governed by the interaction among milling forces, seed hardness, and the heterogeneous organization of starch-, protein-, and cell-wall-rich tissues [6].

For black bean, stone milling shifted a greater proportion of the flour into the 60- and 80-mesh fractions than hammer milling (53.10% vs. 30.60% and 28.75% vs. 2.00%, respectively), whereas hammer milling yielded a substantially greater proportion in the 70-mesh fraction (65.05% vs. 13.25%). Pinto bean exhibited a comparable redistribution, with stone milling producing substantially greater proportions in the 60- and 80-mesh fractions (38.80% and 37.50%, respectively) than hammer milling (24.65% and 16.05%), while hammer milling concentrated more material in the 70-mesh fraction (45.70% vs. 14.65%). Navy bean followed the same general pattern, although the magnitude of the shift differed, with stone milling producing 41.95% and 31.90% in the 60- and 80-mesh fractions, respectively, compared with 24.30% and 6.30% for hammer milling. Collectively, these results suggest that the two milling systems generated different fracture patterns rather than a simple linear difference in flour fineness.

Red kidney bean showed a contrasting response, with similar proportions of flour in the 60-mesh fraction for stone- and hammer-milled samples (28.10% and 29.10%, respectively). A significant difference between milling methods was observed only for this fraction. Significant differences were detected at the 60, 70, and 80 mesh classes for black and pinto beans and at the 60, 70, 80, and 100 mesh classes for navy bean, whereas red kidney bean showed a significant difference only at 60 mesh. This bean-dependent response suggests that the effect of milling cannot be interpreted independently of the structural characteristics of the raw material. Differences in seed hardness, cotyledon structure, and the relative association of starch granules with protein and cell-wall materials can alter how the seed fractures and consequently how material is distributed across particle-size classes [6,9].

Importantly, particle size provides a structural descriptor of the flour but does not fully describe the extent of mechanical modification caused by milling. Milling can simultaneously reduce particle size, disrupt cellular structures, and damage starch granules; therefore, flours with comparable particle-size characteristics may nevertheless differ in starch damage and functional behavior. Previous studies of pulse flours have shown that finer fractions generally exhibit greater starch damage, reflecting the greater mechanical stress required to generate smaller particles [9,10]. Lee et al. [10] further demonstrated that decreasing particle size in pulse flours was accompanied by increased starch damage and changes in pasting and gelling behavior. Thus, the particle-size differences observed in the present study provide an important context for interpreting the damaged-starch results presented subsequently.

The relationship between particle size and functionality is also not necessarily monotonic. Coarse pulse particles can retain aggregated structures containing starch, protein, and dietary fiber, and these particles may contribute disproportionately to flour hydration, pasting, and gelling behavior rather than functioning simply as inert coarse material [10]. Similarly, Bourré et al. [9] showed that particle size influenced water absorption and pasting properties of pulse flours, with the magnitude and direction of the response varying among pulse types. Consequently, the pronounced redistribution observed between the 60–80 mesh fractions in the present study may represent differences in the degree of structural disruption generated by the two milling systems, rather than merely differences in particle diameter.

The overall particle-size distributions did not differ significantly among milling treatments for any bean type (*p* = 0.333). However, mesh-specific comparisons revealed differences within the particle-size profiles, particularly across several adjacent mesh classes for black, pinto, and navy beans. These results suggest that the milling systems produced differences in particle-size distribution patterns for these market classes, although the overall distributions were not significantly different.

Taken together, the results demonstrate that milling method and bean market class jointly determined the particle-size structure of the resulting flours. Stone and hammer milling did not produce a universal “finer” versus “coarser” flour response across all beans; rather, each system generated a characteristic distribution that depended on the structural properties of the bean. This distinction is important for subsequent functional and product evaluations because particle-size distribution can influence starch damage, water interactions, pasting behavior, and ultimately dough and product performance [9,10]. Accordingly, particle-size distribution should be considered together with damaged starch and other flour functionality measurements when interpreting the suitability of stone-milled bean flour for bakery applications.

### 3.3. Damaged Starch

Milling method had a strong effect on damaged starch content, with stone milling producing substantially greater starch damage than hammer milling across all four bean market classes (Table 2). Two-way ANOVA showed significant effects of milling method, F(1, 16) = 5134.83, *p* < 0.001, and bean type, F(3, 16) = 135.31, *p* < 0.001, as well as a significant bean type × milling method interaction, F(3, 16) = 34.67, *p* < 0.001. Stone-milled flours contained 0.820–1.169% damaged starch, whereas corresponding hammer-milled flours contained 0.277–0.400%, representing an approximately 62–66% reduction with hammer milling. Among the stone-milled flours, red kidney bean exhibited the highest damaged starch content (1.169%), followed by navy (1.077%), pinto (0.919%), and black bean (0.820%). These results indicate that starch damage was influenced by both the milling system and the inherent characteristics of the bean market class.

The greater starch damage observed with stone milling is consistent with the mechanical mechanisms involved in dry milling. Compression, shear, impact, and friction can disrupt the physical integrity of starch granules and increase their susceptibility to hydration and enzymatic hydrolysis [20,21]. Under the operating conditions used in the present study, the higher damaged-starch content therefore indicates greater structural disruption during stone milling than during hammer milling. This interpretation is consistent with the direct comparison reported by Pasumarthi et al. [12], in which pinto bean flour produced by stone milling exhibited greater than 1% damaged starch, whereas hammer-milled flour remained below 1%. This agreement supports a milling-method effect under comparable conditions rather than suggesting that stone milling universally produces greater starch damage than hammer milling.

Damaged starch also provides information that cannot be obtained from particle-size distribution alone. Although particle size and starch damage can be related, they describe different aspects of flour structure. Finer pulse flours often exhibit greater starch damage because greater mechanical disruption may be required to generate smaller particles; however, this relationship can vary with pulse type and milling mechanism [9]. In the present study, the large differences in damaged-starch content between milling systems occurred alongside redistribution among the 60–80 mesh fractions rather than a simple shift toward uniformly finer particles. This suggests that the milling treatments differed not only in particle-size reduction but also in the extent to which starch-containing structures were mechanically disrupted. Thus, particle size and damaged starch can be interpreted as complementary descriptors of milling-induced changes in flour structure.

The difference in damaged starch may also have functional implications because damaged starch interacts with water differently from intact starch. Mechanically damaged starch hydrates more readily and is more accessible to amylolytic enzymes, potentially increasing both the amount and rate of water uptake by flour [21,22]. Accordingly, the higher damaged-starch content of the stone-milled flours could increase water requirements during dough preparation and alter the distribution of water among starch, protein, dietary fiber, and other flour components. This may be particularly relevant in pulse–wheat composite systems, where pulse proteins and dietary fiber contribute substantially to water absorption and can modify the development of the wheat gluten network. Previous studies of pulse-enriched wheat doughs have reported increased water absorption and changes in dough development and stability with increasing pulse flour incorporation, reflecting interactions among pulse proteins, dietary fiber, water, and gluten [23]. Thus, even relatively small differences in damaged starch may contribute to differences in flour hydration and dough behavior. The functional implications may extend beyond hydration to dough handling and thermal processing. During mixing, greater water uptake by damaged starch can alter dough consistency and the amount of water available for gluten development. During heating, damaged starch is more readily hydrated and may influence starch swelling, gelatinization, viscosity development, and subsequent structural setting [12]. The direction and magnitude of these effects are product- and process-dependent. In wheat-based systems, high levels of starch damage can increase water absorption and contribute to changes in dough handling, while moderate levels may enhance hydration and contribute to desirable processing characteristics [22,24]. Damaged starch should therefore be considered a functional attribute of flour rather than solely an indicator of milling severity. Nevertheless, these potential effects on dough and product properties were not directly established through a comparison of stone- and hammer-milled flours in the present study.

The variation among bean market classes further suggests that susceptibility to starch damage depends on the structural characteristics of the raw material. Red kidney bean exhibited the highest damaged-starch content after stone milling, whereas black bean exhibited the lowest. Differences in seed hardness and microstructural organization can influence fracture behavior during dry milling and consequently affect the extent to which starch-containing structures are exposed or physically disrupted [6]. This bean-dependent response is consistent with the market-class differences observed in particle-size distribution (Figure 2) and indicates that milling performance reflects the interaction between milling mechanism and the structural characteristics of the bean.

Taken together, the damaged-starch results demonstrate that the two milling systems produced substantially different degrees of starch disruption and that this response varied among bean market classes. The findings also show that particle-size distribution alone does not fully describe the structural changes produced during milling. Differences in damaged starch provide an additional mechanistic basis for understanding potential changes in flour hydration, dough behavior, and thermal processing. Consequently, damaged starch is an important component of the functional characterization of pulse flours and may help explain differences in processing performance associated with milling method and bean market class.

### 3.4. Proximate Composition, Dietary Fiber, and Starch

The proximate composition demonstrated substantial compositional differences between bean flours and wheat flour and smaller but measurable differences among the four bean market classes (Table 3). These differences provide an important compositional context for interpreting the functional behavior of the flour systems.

Dry-matter content ranged from 92.68% for navy bean to 93.37% for black bean, compared with 90.82% for wheat flour. Although these differences were statistically significant, their magnitude was relatively small and is unlikely to be a major determinant of the functional differences observed among the flour systems. Ash content was markedly higher in the bean flours, due to the fact that they are whole grain flour rather than refined flour; ranging from 4.25% to 4.51%, compared with 0.50% in wheat flour. Navy bean had the highest ash concentration (4.51%), followed by black bean (4.36%), pinto bean (4.26%), and red kidney bean (4.25%). The greater ash content of whole-bean flour is consistent with retention of mineral-containing seed components during milling. Because the differences among bean classes were relatively small, ash concentration alone is unlikely to explain the major differences in pasting or product performance. Fat concentration ranged from 1.39% in red kidney bean to 1.97% in navy bean, compared with 1.13% for wheat flour. Although lipids can interact with starch and influence starch functionality, the relatively narrow range among the bean flours suggests that lipid concentration was not the dominant factor determining subsequent pasting behavior [25]. Crude protein showed a much larger nutritional difference between bean and wheat flours. Black bean contained the highest protein concentration (24.90%), followed by pinto (23.89%), red kidney (23.06%), and navy bean (22.96%), compared with 14.97% for wheat flour. These values are consistent with the established high protein content of pulse legumes and support their use as protein-enriching ingredients in cereal-based foods [5]. Pulse proteins can also contribute to hydration, structure formation, and processing behavior.

Dietary fiber concentrations among the bean flours ranged from 17.55% in pinto bean to 18.10% in navy bean. The measured values demonstrate that the bean flours contributed substantial dietary fiber to the formulations. Pulse fibers can influence water retention, texture, and other functional properties of food systems [26]. Starch content ranged from 40.41% in red kidney bean to 44.83% in pinto bean, with no significant difference among the bean types. This relatively narrow range indicates that the large differences observed in pasting behavior were unlikely to result simply from differences in total starch concentration. Instead, the physical state and accessibility of starch, together with protein, dietary fiber, particle-size distribution, and damaged starch, likely contributed more strongly to functional differences. Pulse starches exhibit structural and functional characteristics that differ from cereal starches and are strongly influenced by their non-starch matrix [25].

Taken together, the compositional data establish an important distinction between chemical composition and processing-induced structural modification. Bean types differed in protein and dietary fiber concentrations, whereas milling treatments produced much larger differences in damaged starch. Thus, bean type established the underlying chemical composition of the flour, while milling modified the physical state and accessibility of its components.

### 3.5. Pasting Properties

Pasting properties were substantially modified by the incorporation of bean flour into the wheat-based system (Section 2.4). The response varied among the tested composite flours, indicating that their functional behavior depended on formulation characteristics, including bean market class and substitution level (Table 4).

Peak viscosity (PV) ranged from 426.00 to 545.00 cP among the bean-containing blends, compared with 437.50 cP for the all-purpose flour (APF) control. Navy bean produced the highest PV (545.00 ± 28.28 cP), whereas black bean produced the lowest (426.00 ± 4.24 cP). Pinto bean (436.00 ± 8.49 cP) was numerically similar to the control, whereas red kidney bean produced an intermediate value (472.50 ± 6.36 cP). Differences in PV can reflect the combined effects of starch characteristics, particle size, damaged starch, and interactions with protein and dietary fiber.

Trough viscosity (TV) was lower for all bean-containing blends than for the APF control (217.50 ± 0.71 cP). Values were 165.00 ± 0.00, 187.50 ± 13.44, 150.50 ± 4.95, and 188.50 ± 6.36 cP for black, navy, pinto, and red kidney bean blends, respectively. The lower trough values indicate greater viscosity loss during continued heating and shear than in the APF control. Breakdown viscosity (BD) was consequently greater for all bean-containing blends than for the control. The control exhibited a BD of 220.00 ± 1.41 cP, whereas black, navy, pinto, and red kidney bean blends had values of 261.00 ± 4.24, 357.50 ± 14.85, 285.50 ± 3.54, and 284.00 ± 0.00 cP, respectively. Navy bean had the highest breakdown, indicating that it developed the highest peak viscosity but subsequently experienced the greatest viscosity loss during heating and shear.

The strongest differentiation occurred in final viscosity (FV). The APF control had a final viscosity of 574.50 ± 21.92 cP, whereas the bean-containing blends ranged from 786.50 ± 13.44 cP for black bean to 1301.00 ± 15.56 cP for red kidney bean. The final-viscosity ranking was red kidney > navy > pinto > black bean. The greater final viscosity of the bean-containing systems indicates substantial viscosity development during cooling, which can arise from starch reassociation as well as contributions from non-starch components.

Setback viscosity followed a similar pattern. The APF control had the lowest setback (357.00 ± 22.63 cP), whereas black, navy, pinto, and red kidney bean blends had values of 621.50 ± 13.44, 937.00 ± 24.04, 811.50 ± 64.35, and 1112.50 ± 9.19 cP, respectively. The higher setback values of the bean-containing blends indicate greater increases in viscosity during cooling than in the APF control. Setback viscosity, calculated as final viscosity minus trough viscosity, reflects the combined effects of starch reassociation and changes in paste structure during cooling; however, it is not a direct measurement of molecular retrogradation.

The observed differences may reflect variation in starch characteristics as well as the effects of protein and non-starch polysaccharides on water distribution and paste consistency [25,26]. Starch molecular characteristics, including amylose content and amylopectin structure, can influence granule swelling, pasting behavior, and viscosity development during cooling. Amylose reassociation can contribute to early paste firming, whereas amylopectin reassociation is generally more relevant to structural changes during subsequent storage [25]. Differences in these characteristics may therefore have contributed to the variation in final and setback viscosities among the composite flours. However, amylose/amylopectin ratios and starch molecular characteristics were not determined in the present study, so their individual contributions cannot be established.

Non-starch polysaccharides may also influence the pasting behavior of pulse-containing flours. Pulse cotyledon cell walls contain pectin and other structural polysaccharides, while starch granules are embedded in a protein-containing cellular matrix. These structures can affect water distribution and restrict starch-granule swelling; soluble polysaccharide fractions may also contribute to water binding and the viscosity of the continuous phase [26,27]. Differences in the amount, solubility, and physical distribution of cell-wall material could consequently contribute to the distinct viscosity profiles. Nevertheless, pectin concentration, molecular structure, solubilization, and cell-wall integrity were not measured in this study. Their contribution should therefore be considered a plausible mechanism rather than a demonstrated cause of the observed differences.

Bean proteins may further influence starch pasting by competing for water, affecting granule swelling, and contributing to the structure of the composite flour matrix during heating and cooling. The functional properties of pulse proteins, including their water-binding and aggregation behavior, can affect the performance of pulse-containing food systems [5]. Protein–starch interactions may also modify the rheological behavior of starch-containing systems during cooling, although their effects depend on composition and processing conditions [27]. These interactions may have contributed to the differences in final and setback viscosities observed here, but protein solubility, protein aggregation, and molecular-level protein–starch interactions were not directly characterized. Therefore, the higher final and setback viscosities of the red kidney bean blend cannot be attributed to a specific protein–starch interaction.

The RVA results should also be interpreted in relation to particle size and damaged starch rather than as isolated measurements. Previous work has demonstrated that finer pulse flours can exhibit greater starch damage and higher peak and final viscosities, while coarse particles containing aggregated starch, protein, and dietary fiber can also contribute substantially to pasting behavior [9,10]. The present results are consistent with the possibility that differences in flour structure and composition contributed to the distinct viscosity profiles. However, the observed RVA differences cannot be attributed to damaged starch alone because the bean flours also differed in protein and fiber composition and particle-size characteristics. This interpretation is consistent with Pasumarthi et al. [12], who demonstrated that milling-induced changes in pinto bean starch damage and particle characteristics affected the functional properties of the resulting flour.

Importantly, the RVA measurements were conducted using stone-milled bean flours only. Furthermore, the black bean blend contained 30% bean flour, whereas the navy, pinto, and red kidney bean blends contained 40%. Differences in both bean market class and substitution level may therefore have contributed to the observed RVA profiles, and their individual effects cannot be separated in the present experimental design. Accordingly, the RVA results represent the integrated response of each composite flour to heating, shear, and cooling rather than direct measurements of starch retrogradation, pectin integrity, or protein–starch network formation. They also do not establish differences in pasting behavior between stone- and hammer-milled flours.

Overall, incorporation of dry bean flour substantially increased final and setback viscosities relative to APF, while the magnitude of the response varied among the tested composite flours. Navy bean generated the highest peak and breakdown viscosities, whereas the red kidney bean blend produced the highest final and setback viscosities. These findings demonstrate distinct pasting profiles among the tested stone-milled bean–wheat blends. Further investigation using a common bean substitution level and direct characterization of starch structure, cell-wall polysaccharides, and protein functionality would help clarify the mechanisms underlying these differences.

### 3.6. Product Development

**Figure 3:**
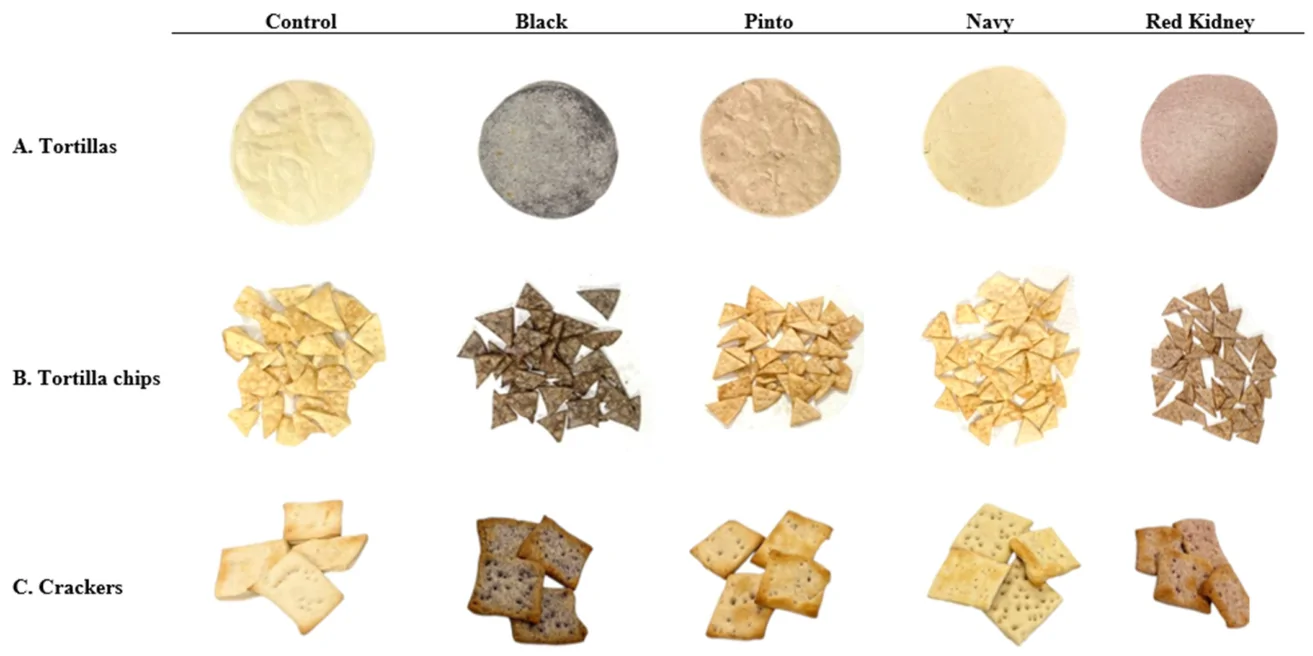
Representative appearance of bakery products prepared from bean–wheat composite flour blends. Representative images of tortillas, tortilla chips, and crackers prepared using bean–wheat composite flour blends. Products were prepared using the selected stone-milled bean flour incorporation levels described in the Section 2 Materials and Methods.

Products were developed with 30% (*w*/*w*) black bean flour and 40% (*w*/*w*) pinto, navy, and red kidney bean flour as the final substitution levels for preparation of tortillas, tortilla chips, and crackers (Figure 3).

### 3.7. Sensory Evaluation of Crackers, Tortillas, and Tortilla Chips

**Figure 4:**
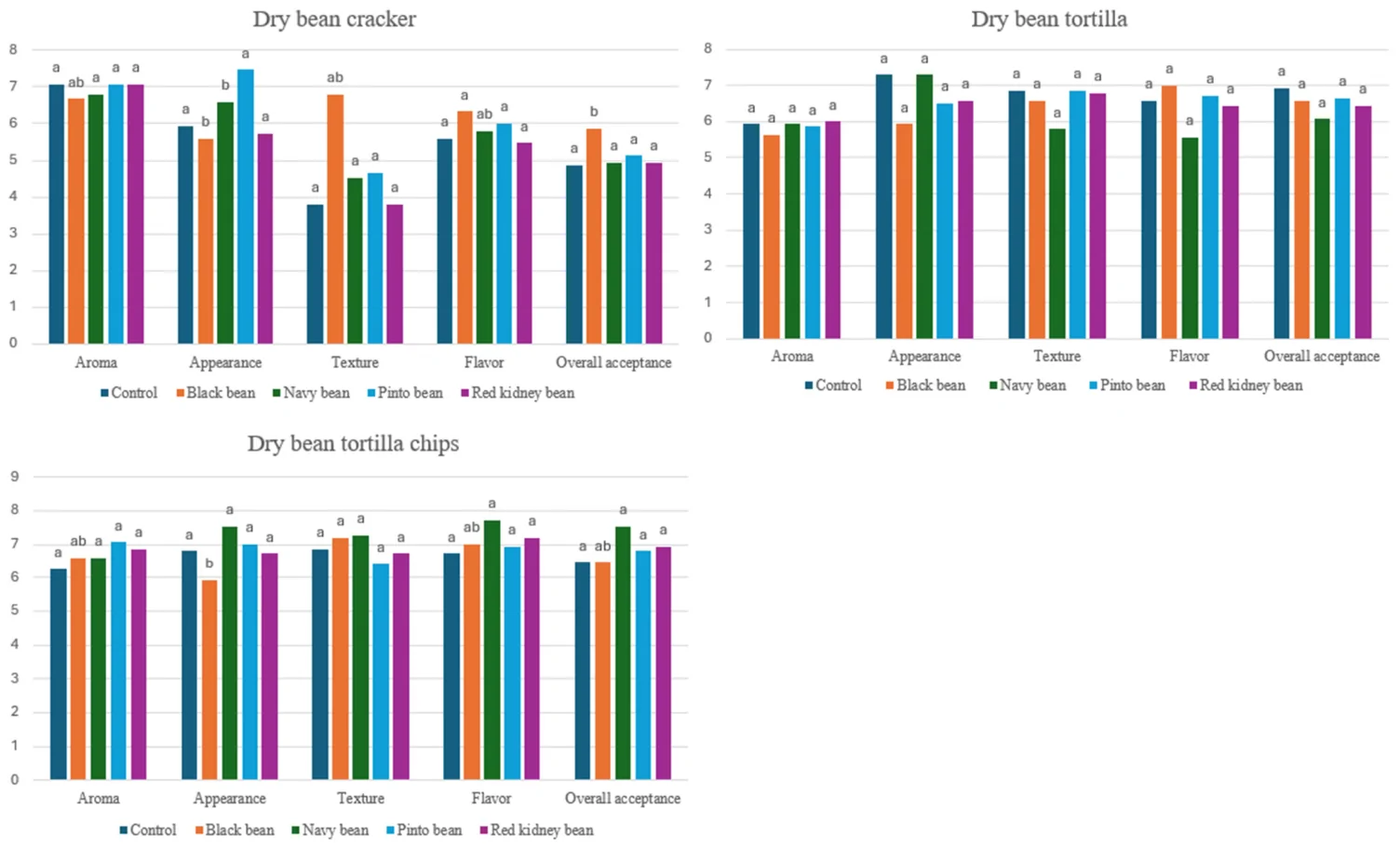
Sensory evaluation of dry bean crackers, tortilla and tortilla chips, based on aroma, appearance, texture, flavor, and overall acceptance. Mean sensory liking scores for sensory attributes of bakery product with incorporation of different bean flour sources. Values are means ± standard deviation (SD) (*n* = 15). Different lowercase letters indicate significant differences among formulations (*p* < 0.05), based on repeated-measures ANOVA followed by Holm-adjusted paired comparisons.

Sensory evaluation was conducted with 15 panelists for each product category, and formulation effects were evaluated separately for crackers, tortillas, and tortilla chips (Figure 4). Scores were interpreted on the 9-point hedonic scale, where scores around 5 indicate a neutral level of liking and higher scores indicate greater acceptability. The influence of bean incorporation was product-dependent, with the strongest formulation effects observed for crackers and more limited effects for tortillas and tortilla chips.

#### 3.7.1. Crackers

Cracker formulation significantly affected appearance and texture, whereas aroma, flavor, and overall acceptance did not differ significantly among formulations. Appearance differed significantly among formulations, F(4, 56) = 6.493, *p* < 0.001. Pinto bean crackers received the highest appearance score (7.47), significantly exceeding the control (5.93), black bean (5.60), and red kidney bean (5.73) crackers. Navy bean crackers had an intermediate appearance score of 6.60. Texture was also significantly affected by formulation, F(4, 56) = 12.477, *p* < 0.001. Black bean crackers received the highest texture score (6.80), significantly exceeding the control (3.80), navy bean (4.53), and red kidney bean (3.80) crackers. Pinto bean crackers had an intermediate score of 4.67. In contrast, aroma, F(4, 56) = 0.471, *p* = 0.757, and flavor, F(4, 56) = 1.559, *p* = 0.198, were not significantly affected by formulation. The absence of a significant flavor response indicates that, under the formulation conditions used, bean incorporation did not produce detectable differences in flavor liking among the tested products. Overall acceptance also did not differ significantly among cracker formulations, F(4, 56) = 1.236, *p* = 0.306. Mean scores were 4.87 for the control, 5.87 for black bean, 4.93 for navy bean, 5.13 for pinto bean, and 4.93 for red kidney bean. Thus, although bean incorporation significantly changed specific sensory attributes, particularly appearance and texture, these differences did not result in a statistically significant difference in overall acceptance.

#### 3.7.2. Tortillas

In contrast to crackers, tortilla formulation did not significantly affect any evaluated sensory attribute. Aroma scores ranged from 5.64 for black bean to 6.00 for red kidney bean, with no significant formulation effect, F(4, 56) = 0.220, *p* = 0.926. Appearance also did not differ significantly, F(4, 56) = 2.132, *p* = 0.090, despite numerical differences among formulations.

Texture was not significantly affected by formulation, F(4, 56) = 1.607, *p* = 0.187. The control and pinto bean tortillas had the highest numerical texture scores (6.86), whereas navy bean tortillas had the lowest (5.79). Flavor likewise did not differ significantly, F(4, 56) = 2.123, *p* = 0.091. Overall acceptance was also statistically comparable among formulations, F(4, 56) = 0.705, *p* = 0.592. The control had the highest numerical score (6.93), followed by pinto (6.64), black (6.57), red kidney (6.43), and navy bean (6.07) tortillas. These results indicate that incorporation of the different bean flours did not significantly compromise sensory acceptance of the tortillas.

#### 3.7.3. Tortilla Chips

For tortilla chips, formulation significantly affected appearance, F(4, 56) = 3.448, *p* = 0.014, whereas aroma, texture, and flavor were not significantly affected. Navy bean chips received the highest appearance score (7.53), whereas black bean chips received the lowest (5.93). A significant difference between the navy and black bean formulations was observed, whereas the other comparisons were not significant. Aroma, F(4, 56) = 1.149, *p* = 0.343, texture, F(4, 56) = 1.491, *p* = 0.217, and flavor, F(4, 56) = 2.019, *p* = 0.104, did not differ significantly among formulations. Navy bean chips nevertheless had the highest numerical texture and flavor scores, at 7.27 and 7.73, respectively. Overall acceptance differed significantly among treatments, F(4, 56) = 2.766, *p* = 0.036. Navy bean chips had the highest numerical overall acceptance score (7.53), followed by red kidney (6.93), pinto (6.80), and the control and black bean formulations (both 6.47). However, no significant pairwise differences were detected among treatments.

#### 3.7.4. Integrated Sensory Interpretation

The sensory response to bean flour was strongly dependent on the product matrix, but incorporation did not consistently reduce acceptability despite the sensory challenges often associated with pulse ingredients. Tortilla chips showed the most favorable overall response, particularly for navy bean, which received the highest numerical scores for appearance (7.53), texture (7.27), flavor (7.73), and overall acceptance (7.53). Although overall acceptance did not differ significantly among formulations in pairwise comparisons, the consistently high scores across attributes indicate good sensory compatibility of navy bean flour in this product.

Crackers also showed potential for bean flour incorporation, with black bean receiving the highest texture (6.80) and overall acceptance (5.87) scores and pinto bean receiving the highest appearance score (7.47). Tortillas showed no significant differences among bean types for any sensory attribute, suggesting that this product matrix can accommodate different bean flours without substantial changes in sensory acceptance. Overall, the results identify navy bean for tortilla chips and pinto and black bean for crackers as promising candidates for further product development, based on their favorable sensory profiles and absence of major sensory penalties. These findings support further optimization of bean flour concentration, milling characteristics, and processing conditions to develop value-added products that retain desirable sensory quality while incorporating the nutritional and functional benefits of bean flour.

### 3.8. Exploratory Relationships Among Flour Characteristics, Pasting Properties, and Sensory Responses

Exploratory Spearman correlation analysis was conducted to examine associations among particle-size distribution, damaged starch, pasting properties, and sensory responses (Supplementary Table S2). Across the eight-bean class × milling method combinations, no statistically significant correlations were observed between individual particle-size fractions and damaged starch (*p* > 0.05), suggesting that particle-size distribution alone did not consistently explain starch damage across milling systems. Among the four stone-milled bean formulations, damaged starch showed strong positive rank associations with final viscosity and setback (ρ = 1.00 for both). Relationships with sensory attributes varied among product matrices. Cracker texture exhibited a negative rank association with damaged starch and final viscosity (ρ = −1.00), whereas tortilla-chip overall acceptance showed a positive association with peak viscosity (ρ = 1.00). Comparable relationships were less consistent for tortillas. These observations suggest that associations between flour characteristics, pasting behavior, and sensory responses may depend on the product matrix and processing conditions. However, the correlations involving RVA and sensory attributes were based on only four bean-containing formulations, with black bean flour incorporated at 30% compared with 40% for the other bean classes. Consequently, these relationships should be interpreted as exploratory rather than causal or predictive. Additional studies using standardized incorporation levels and larger numbers of independent formulations are needed to establish these associations.

### 3.9. Protein, Dietary Fiber, and Amino Acid Composition of Pinto Bean-Based Bakery Products

Pinto bean flour contained 23.89% protein and 17.55% dietary fiber (Table 5). Following incorporation into the bakery formulations, protein concentrations were 16.37% in the cracker, 15.90% in the chips, and 14.07% in the tortilla. The cracker and chips contained significantly greater protein concentrations than the tortilla (*p* < 0.05), whereas all three products contained less protein than the original bean flour. Dietary fiber showed a similar pattern, decreasing from 17.55% in the flour to 8.07% in the cracker, 7.43% in the chips, and 6.73% in the tortilla. The cracker contained significantly more dietary fiber than the chips and tortilla, while the latter two products did not differ significantly. These differences reflect the composition of the final product formulations, in which pinto bean flour was combined with refined wheat flour and other ingredients, rather than indicating nutrient losses during processing.

The relatively high protein and dietary fiber concentrations retained in the finished products demonstrate the nutritional contribution of pinto bean flour to the composite bakery formulations. Pulses are recognized as important sources of plant protein and dietary fiber, and incorporation of pulse flours into cereal-based foods can increase the concentration of these nutrients relative to products formulated predominantly with refined cereal flour [5]. The differences among the three products further indicate that the nutritional contribution of the bean ingredient was influenced by product formulation and processing format. In particular, the higher protein and dietary fiber concentrations measured in the cracker indicate greater nutrient density on a product-weight basis than in the chips and tortilla. The amino-acid measurements provide additional descriptive information on the protein composition of the products. Pinto bean flour contained 70.00 mg/g essential amino acids (EAA), 33.00 mg/g branched-chain amino acids (BCAA) and 6.00 mg/g sulfur-containing amino acids (SAA). Corresponding values in the finished products were 47.45, 38.73, and 34.55 mg/g EAA for the cracker, chips, and tortilla, respectively; 23.39, 19.00, and 17.31 mg/g BCAA; and 4.67, 3.86, and 3.38 mg/g SAA. These values indicate that the pinto bean ingredient contributed measurable amounts of essential amino acids to each bakery product. However, because the amino-acid concentrations were determined from single analytical measurements and are expressed on a sample basis, they are presented as descriptive compositional values rather than as statistically compared outcomes or measures of amino-acid retention.

The relative contribution of the different amino-acid groups is consistent with the general nutritional characteristics of pulse proteins. Pulse proteins provide appreciable amounts of essential amino acids, particularly lysine and branched-chain amino acids, but sulfur-containing amino acids are generally present at lower concentrations [5]. The present results show the same general pattern, with SAA concentrations lower than the corresponding EAA and BCAA concentrations in both the bean flour and finished products. This characteristic is relevant to pulse–cereal composite foods because cereal and pulse proteins have complementary amino-acid patterns, making their combination nutritionally relevant even though the present data do not permit calculation of protein quality or amino-acid adequacy.

Overall, the results demonstrate that pinto bean flour contributed substantial protein and dietary fiber to the bakery formulations while also providing essential amino acids in the finished products. Among the products evaluated, the cracker exhibited the highest protein and dietary fiber concentrations and the highest measured EAA, BCAA, and SAA concentrations on a sample basis. The tortilla exhibited the lowest values for these compositional measures. These differences should be interpreted primarily as differences in the composition and nutrient density of the final product formats rather than as evidence of differential amino-acid stability during processing.

## 4. Conclusions

This study demonstrated that milling method and dry bean market class influenced flour particle-size distribution and starch damage. Stone milling produced a more uniform particle-size distribution and greater starch damage than hammer milling, although the magnitude of these effects varied among bean classes.

In product applications, stone-milled dry bean flours influenced the pasting properties of wheat-based flour blends and the sensory characteristics of tortillas, tortilla chips, and crackers, with responses varying according to bean class, product matrix, and processing conditions. Favorable sensory responses in selected bean flour–product combinations demonstrated the potential to incorporate stone-milled dry bean flours into wheat-based foods while maintaining sensory acceptability. Pinto bean flour also contributed protein and dietary fiber to the developed products.

As a proof of concept, these findings support the potential application of stone-milled dry bean flours in selected wheat-based products. However, because only stone-milled flours were evaluated in product development, the effects of milling method on finished-product quality could not be directly established. Furthermore, the findings are limited to the milling conditions, bean classes, and formulations investigated and do not establish optimal processing parameters or predict performance across broader applications. Future research should directly compare stone- and hammer-milled flours in equivalent product formulations and investigate relationships among milling conditions, flour functionality, processing efficiency, and finished-product quality.

## Figures and Tables

**Table 1:** Color characteristics (L*, a*, and b*) of stone- and hammer-milled flour from four *Phaseolus vulgaris* L. market classes (black, navy, pinto, and red kidney bean).

Sample	Milling Method	L* (Lightness)	a* (Redness/Greenness)	b* (Yellowness/Blueness)
Black bean	Stone	24.93 ± 0.26 ^c^	1.49 ± 0.09 ^d^	−0.20 ± 0.57 ^f^
	Hammer	25.11 ± 0.40 ^c^	0.97 ± 0.19 ^d^	−0.72 ± 0.19 ^f^
Navy bean	Stone	68.73 ± 1.76 ^a^	2.53 ± 0.03 ^d^	11.05 ± 0.18 ^c^
	Hammer	67.99 ± 0.52 ^a^	2.64 ± 0.03 ^d^	11.48 ± 0.12 ^c^
Pinto bean	Stone	52.42 ± 1.47 ^b^	6.97 ± 0.50 ^c^	14.35 ± 0.10 ^a^^b^
	Hammer	52.68 ± 0.60 ^b^	8.41 ± 0.21 ^c^	15.54 ± 0.11 ^a^
Red kidney bean	Stone	28.81 ± 0.40 ^c^	19.46 ± 0.95 ^a^	5.80 ± 0.32 ^d^
	Hammer	30.45 ± 0.88 ^c^	18.64 ± 0.57 ^b^	5.95 ± 0.13 ^d^

Values are presented as mean ± standard deviation (*n* = 3). Different superscript letters within the same column indicate significant differences among treatments (*p* < 0.01).

**Table 2:** Damaged starch content of stone- and hammer-milled bean flours from four *Phaseolus vulgaris* L. market classes (black, navy, pinto, and red kidney bean).

Bean Type	Stone Milling	Hammer Milling
Black bean	0.820 ± 0.050 ^c^	0.277 ± 0.005 ^d^
Navy bean	1.077 ± 0.004 ^e^	0.382 ± 0.005 ^f^
Pinto bean	0.919 ± 0.031 ^a^	0.348 ± 0.006 ^b^
Red kidney bean	1.169 ± 0.018 ^g^	0.400 ± 0.001 ^h^

Values are presented as mean ± standard deviation (SD) (*n* = 3). Data were analyzed by two-way analysis of variance (ANOVA) with milling method and bean type as fixed factors. The milling method combined by bean type interaction was significant (*p* < 0.001). Different superscript lowercase letters indicate significant differences among the eight bean type combined by milling method according to Tukey’s honestly significant difference (HSD) test (*p* < 0.05).

**Table 3:** Proximate composition, total starch, and dietary fiber content of bean flours and refined wheat flour on a dry-matter basis.

Sample	Dry Matter	Ash	Fat (EE)	Crude Protein	Dietary Fiber	Starch
Wheat flour	90.82 ± 0.02 ^d^	0.50 ± 0.02 ^c^	1.13 ± 0.01 ^e^	14.97 ± 0.04 ^d^	ND	ND
Black bean flour	93.37 ± 0.06 ^a^	4.36 ± 0.05 ^a^	1.57 ± 0.01 ^b^	24.90 ± 0.03 ^a^	17.58 ± 0.95 ^a^	41.03 ± 2.11 ^a^
Navy bean flour	92.68 ± 0.01 ^c^	4.51 ± 0.04 ^a^	1.97 ± 0.02 ^a^	22.96 ± 0.07 ^c^	18.10 ± 0.47 ^a^	41.83 ± 2.51 ^a^
Pinto bean flour	93.01 ± 0.01 ^b^	4.26 ± 0.10 ^b^	1.49 ± 0.00 ^c^	23.89 ± 0.03 ^b^	17.55 ± 0.07 ^a^	44.83 ± 0.95 ^a^
Red kidney bean flour	93.22 ± 0.14 ^a^	4.25 ± 0.07 ^b^	1.39 ± 0.00 ^d^	23.06 ± 0.06 ^c^	17.77 ± 0.30 ^a^	40.41 ± 2.55 ^a^

Values are presented as mean ± standard deviation (SD) *(n* = 3). Different superscript lowercase letters within the same column indicate significant differences among samples (*p* < 0.05). ND = not determined; EE = ether extract. Crude protein was calculated as nitrogen × 6.25. Values for dietary fiber and total starch were determined by an external analytical laboratory using standardized analytical procedures and are reported on a dry-matter basis.

**Table 4:** Pasting properties of refined wheat flour and bean–wheat composite flour blends determined by Rapid Visco Analyzer.

Sample	Peak Viscosity (PV, cP)	Trough Viscosity (TV, cP)	Breakdown Viscosity (BD, cP)	Final Viscosity (FV, cP)	Setback Viscosity (SB, cP)
Control (APF)	437.50 ± 0.71 ^b^	217.50 ± 0.71 ^a^	220.00 ± 1.41 ^b^	574.50 ± 21.92 ^e^	357.00 ± 22.63 ^d^
Black bean (30%)	426.00 ± 4.24 ^b^	165.00 ± 0.00 ^b^^c^	261.00 ± 4.24 ^c^	786.50 ± 13.44 ^d^	621.50 ± 13.44 ^c^
Navy bean (40%)	545.00 ± 28.28 ^a^	187.50 ± 13.44 ^b^	357.50 ± 14.85 ^a^	1124.50 ± 37.48 ^b^	937.00 ± 24.04 ^b^
Pinto bean (40%)	436.00 ± 8.49 ^b^	150.50 ± 4.95 ^c^	285.50 ± 3.54 ^b^^c^	962.00 ± 69.30 ^c^	811.50 ± 64.35 ^b^
Red kidney bean (40%)	472.50 ± 6.36 ^a^^b^	188.50 ± 6.36 ^b^	284.00 ± 0.00 ^b^^c^	1301.00 ± 15.56 ^a^	1112.50 ± 9.19 ^a^

Values are presented as mean ± standard deviation (SD) (*n* = 2). Different superscript lowercase letters within the same column indicate significant differences among samples according to Tukey’s honestly significant difference (HSD) test (*p* < 0.05). APF = all-purpose flour. Values in parentheses indicate the percentage of bean flour incorporated into the composite flour blend. PV = peak viscosity; TV = trough viscosity; BD = breakdown viscosity; FV = final viscosity; SB = setback viscosity.

**Table 5:** Protein, dietary fiber, and selected amino acid characteristics of pinto bean flour and pinto bean-based bakery products.

Sample	Protein (%)	Dietary Fiber (%)	EAA (mg/g)	BCAA (mg/g)	SAA (mg/g)
Pinto bean flour	23.89 ± 0.10 ^a^	17.55 ± 0.10 ^a^	70.00	33.00	6.00
Pinto bean cracker	16.37 ± 0.76 ^b^	8.07 ± 0.40 ^b^	47.45	23.39	4.67
Pinto bean chips	15.90 ± 0.10 ^b^	7.43 ± 0.40 ^c^	38.73	19.00	3.86
Pinto bean tortilla	14.07 ± 0.40 ^c^	6.73 ± 0.25 ^c^	34.55	17.31	3.38

Protein and dietary fiber values are presented as mean ± standard deviation (SD) (*n* = 3). Different superscript lowercase letters within the same column indicate significant differences among samples according to Tukey’s honestly significant difference (HSD) test (*p* < 0.05). EAA = essential amino acids; BCAA = branched-chain amino acids; SAA = sulfur-containing amino acids. Amino acid concentrations are expressed as mg/g sample and represent single analytical measurements (*n* = 1); therefore, standard deviations and statistical comparisons were not calculated. EAA includes threonine, valine, isoleucine, leucine, phenylalanine, lysine, histidine, and methionine; BCAA includes valine, isoleucine, and leucine; SAA includes methionine and cystine.

## Data Availability

The original contributions presented in this study are included in the article/supplementary material. Further inquiries can be directed to the corresponding author.

## Supplementary Materials

The following supporting information can be downloaded at: https://www.mdpi.com/article/10.3390/foods15214002/s1, Table S1: Individual amino acid composition of pinto bean-based bakery products; Table S2: Selected exploratory Spearman rank correlations among damaged starch, RVA pasting parameters, and sensory attributes of stone-milled dry bean flour products.
