# Supplementary material for: Stone Milling of Dry Beans: Comparison with Hammer Milling, Physicochemical Characteristics of Flour, and Application in Wheat-Based Bakery Products
Source: Foods. 2026 Nov 7;15(21):4002. doi: 10.3390/foods15214002 (PMC13762995; doi:10.3390/foods15214002)
Supplement: Supplementary file 1 [file foods-15-04002-s001.zip › foods-4639335-supplementary.pdf]

foods

Supplementary Material

Supplementary Table S1. Individual amino acid composition of pinto bean-based bakery products.

Amino acid	Pinto bean cracker (mg/g)	Pinto bean chips (mg/g)	Pinto bean tortilla (mg/g)
Aspartic acid	11.20	9.27	8.21
Threonine	5.01	4.13	3.76
Serine	7.47	6.17	5.46
Glutamic acid	35.10	27.60	26.00
Proline	11.00	8.66	7.90
Glycine	5.45	4.35	3.94
Alanine	5.33	4.28	3.96
Valine	6.84	5.51	4.99
Isoleucine	5.95	4.85	4.41
Leucine	10.60	8.64	7.91
Tyrosine	5.28	4.22	3.85
Phenylalanine	7.70	6.27	5.72
Lysine	5.73	4.71	4.75
Histidine	3.45	2.82	2.51
Arginine	7.45	6.10	5.39
Cystine	2.50	2.06	1.73
Methionine	2.17	1.80	1.65

Values are reported as mg/g sample basis. Analytical values represent single measurements; therefore, standard deviation and statistical comparisons were not calculated. Essential amino acids include threonine, valine, isoleucine, leucine, phenylalanine, lysine, histidine, and methionine. Branched-chain amino acids include valine, isoleucine, and leucine. Sulfur-containing amino acids include methionine and cystine.

Supplementary Table S2. Selected exploratory Spearman rank correlations among damaged starch, RVA pasting parameters, and sensory attributes of stone-milled dry bean flour products.

Variable 1	Variable 2	Spearman correlation coefficient (ρ)
Damaged starch	Final viscosity (FV)	1.00
Damaged starch	Setback viscosity (SB)	1.00
Damaged starch	Cracker texture	−1.00
Final viscosity (FV)	Cracker texture	−1.00
Peak viscosity (PV)	Tortilla chip overall acceptance	1.00
Damaged starch	Tortilla overall acceptance	−0.60

Correlations were calculated using treatment means for four stone-milled bean flour formulations (black, navy, pinto, and red kidney beans; n = 4). RVA parameters were measured in bean–wheat flour blends, and sensory attributes were represented by mean panel scores for each formulation. Black bean flour was incorporated at 30%, whereas the other bean flours were incorporated at 40%. The wheat-only control was excluded. Correlation coefficients are presented for exploratory purposes because of the limited number of independent formulations. These associations should not be interpreted as evidence of causation or predictive relationships.
